# Overexpression of JAB1 promotes malignant behavior and predicts poor prognosis in esophageal squamous cell carcinoma

**DOI:** 10.1111/1759-7714.13350

**Published:** 2020-02-16

**Authors:** Qi Shen, Bin Shang, Bin Jiang, Yu Wang, Zhou Wang, Gang Chen

**Affiliations:** ^1^ Department of Thoracic Surgery Shandong Provincial Hospital Affiliated to Shandong University Jinan China

**Keywords:** Esophageal squamous cell carcinoma, JAB1, prognosis

## Abstract

**Background:**

This study investigated the expression and biological function of JAB1 in esophageal squamous cell carcinoma (ESCC).

**Methods:**

The expression of JAB1 in ESCC tissues and cells was measured using reverse transcriptase‐polymerase chain reaction (RT‐PCR), immunohistochemistry (IHC), and western blot analysis. Kaplan‐Meier survival analysis was performed to explore the effect of JAB1 expression on the prognosis of ESCC patients. Furthermore, experiments were conducted in vivo and in vitro to determine the effect of JAB1 expression on the malignant behavior of ESCC cells.

**Results:**

Compared with adjacent tissues, JAB1 was highly overexpressed in cancer tissues (*P* = 0.01). Univariate and multivariate analyses of clinical data indicated that patients with JAB1 overexpression had a worse prognosis (*P* = 0.001 and *P* = 0.049, respectively). Cell function experiments and tumorigenesis experiments in nude mice showed that the upregulation of JAB1 might promote malignant behavior, and vice versa.

**Conclusions:**

Overexpression of JAB1 promoted the proliferation, migration, and invasion of ESCC cells, and was significantly associated with poor prognosis of ESCC patients. Therefore, JAB1 could be considered as a promising prognostic factor and a possible target for the specific therapy of ESCC.

**Key points:**

In this study, we found that JAB1 was highly overexpressed in cancer tissues, which could influence the malignant behavior of ESCC cells, and was significantly associated with poor prognosis of ESCC patients.

## Introduction

As one of the most fatal malignant tumors of the digestive tract, esophageal cancer was reported to be the seventh most common cancer in the world in 2018. Each year, approximately 500 000 people worldwide are diagnosed with esophageal cancer, and over 400 000 of the world's population die of esophageal cancer and relevant complications.^1^, ^2^ China currently ranks the fifth highest in the world for the incidence of esophageal cancer and accounts for over 50% of new esophageal cancer cases worldwide. Esophageal squamous cell carcinoma (ESCC) is the predominant pathological subtype of esophageal cancer in China. ESCC is approximately three times more common in men than in women, which is probably related to the unhealthy lifestyle (e.g., smoking, drinking) adopted by Chinese males.^3^, ^4^

Surgical intervention has been reported to be the mainstay of treatment for patients with early‐stage ESCC when the disease is confined to the esophagus.^5^ However, the overall prognosis is poor. Lymph node metastasis during the earlier stages is one of the essential factors that affect the prognosis in ESCC patients.^6^ In many cases, recurrence or metastasis has been reported within three years following surgery. There is a range of causes of lymph node metastasis in ESCC patients. Anatomically, the mucosa of the esophagus contains lymphatic vessels, and as such, lymph node metastasis may occur when the tumor is confined to the mucosa, although the tumor‐node‐metastasis (TNM) is still in the T1 stage.^7^, ^8^ Several molecular markers play an essential role in the metastasis of ESCC cells. Notably, it is believed that the epithelial–mesenchymal transition (EMT) is an essential process during the metastasis of ESCC.^9^, ^10^, ^11^ The expression of EMT indicator proteins, to some extent, reflects the metastatic ability of ESCC cells.^12^, ^13^, ^14^, ^15^, ^16^

JAB1 is an evolutionarily conserved and multifunctional protein that is involved in ubiquitin‐mediated protein degradation; it was initially identified as c‐Jun activation domain‐binding protein‐1 and subsequently discovered to be the fifth component of the constitutive photomorphogenic‐9 signalosome (COPS5, CSN5). JAB1 interacts with and controls multiple intracellular signaling molecules such as c‐Jun, p27, HIF1α, Smad4, p53, and CUL1 (SCF) in mammalian cells.^17^, ^18^

Substantial evidence has accumulated showing that JAB1 is an oncoprotein. As previously reported by Wang and Tsai, JAB1 is a proto‐oncogene that shows abnormal expression in a variety of human cancers and plays an essential role in the development and metastasis of cancers; a high level of JAB1 is often correlated with a poor prognosis.^17^, ^18^ Knockdown of JAB1 inhibits the proliferation of human tumor cells, suggesting that overexpression of JAB1 not only serves as a marker of malignant transformation, but also actually contributes to tumor cell proliferation. Nevertheless, only a limited number of reports discuss the role of JAB1 in the development and progression of ESCC, and the relevant mechanisms remain unclear.

This study investigated the expression of JAB1 in ESCC and explored its effect on proliferation, invasion, and metastasis of ESCC, and the related molecular mechanisms, as well as its effect on the prognosis of ESCC patients.

## Methods

### Ethical approval

The Institutional Ethics Committee of Provincial Hospital affiliated with Shandong University approved this research program in accordance with the ethical guidelines of the Declaration of Helsinki and the guidelines of the China Ethics Review Committee (LCYJ: NO.2019‐162). Written informed consent was provided by all patients enrolled in the study.

The animal assay protocol was approved by the Institutional Ethics Committee of the Provincial Hospital affiliated with Shandong University (No. 2019‐006). All operations involved in the assay were performed in strict accordance with the applicable laws and regulations of the People's Republic of China (Regulations for the Administration of Affairs Concerning Experimental Animals).

### Patients

This study included 30 groups of frozen tissue samples (ESCC tissue and normal tissue adjacent to the tumor [NAT] of the mucous membrane of the esophagus, 5 cm from the tumor) collected from patients who were admitted to Provincial Hospital affiliated with Shandong University and diagnosed with ESCC during 2016 and 2017. Additionally, there were 124 groups of formalin‐fixed paraffin‐embedded ESCC tissue samples collected between 2010 and 2013. Inclusion criteria were as follows: (i) confirmation of stage I to III ESCC based on pathological diagnosis without apparent distant metastasis or surgical contraindications before operation; (ii) no preoperative chemotherapy or radiotherapy; (iii) no contraindications to surgery, complete removal of the tumor in the Ivor Lewis esophagectomy, with the margins of resection of 5 cm or above and elimination of residual tumor cells, and performance of two‐field lymph node dissection; (iv) participation in the five‐year clinical follow‐up program, with the date of the last follow‐up being recorded; (v) Postoperative TNM staging according to the TNM staging (2009) system established by the International Union Against Cancer. Written informed consent was provided by all patients enrolled in the study. The institutional ethics committee approved this research program of Provincial Hospital affiliated with Shandong University in accordance with the guidelines of the China Ethics Review Committee.

### Cell culture and transfection

Four human ESCC cell lines (including Eca109, KYSE150, KYSE450, and EC9706) and the HET‐1A cell line were purchased from the Cell Bank of the Shanghai Institute in China and used in the experiments involved in this study. RPMI‐1640 containing fetal bovine serum (FBS) (Thermo Fisher Scientific) was used for cell culture. An overexpression vector for the human JAB1 gene was prepared through chemical synthesis and encapsulated into a lentivirus (Genechem, Shanghai, China). Polybrene (6 μg/mL) (Genechem, Shanghai, China) was used for the enhancement of lentiviral transfection efficiency. Each group had cells transfected with control sequences (overexpression control) and with target sequences (OEJAB1), respectively. Culture medium (100 μL), the lentivirus (50:1 multiplicity of infection [MOI]), and the transfection reagent were successively added to a 96‐well plate, and the medium was replaced after transfection for eight hours. The cells were observed with a high‐content microscope, and when they had been transfected with green fluorescent protein (GFP), puromycin (6 μg/mL) was added for screening that lasted a week. Three small interfering RNAs (siRNAs) containing different sequences were obtained via chemical synthesis and labeled as SiJAB1‐1, SiJAB1‐2, and SiJAB1‐3. A six‐well culture plate was used for cell culture until the cells were approximately 40% confluent. The cells were then divided into four groups, including a control group siControl and the other three groups that were transfected with SiJAB1‐1, SiJAB1‐2, and SiJAB1‐3, respectively. The culture medium was replaced with fresh medium after transfection for eight hours. Proteins were extracted 24 hours later to assess the transfection efficiency, and the cells transfected successfully were subsequently used for experiments.

### Immunohistochemistry (IHC) staining and assessment

The conventional streptomycin‐horseradish peroxidase (HRP) method was employed in the evaluation of JAB1 expression in tissue samples. Tissue samples were diluted at the volume ratio of 1:500 with the primary antibody, labeled with an HRP conjugated secondary antibody (Zhongshan Jinqiao Biotechnology, China) and then stained with 3,3′‐diaminobenzidine. Two independent pathologists completed the IHC assessment, and the patients were divided into two groups, namely a high‐expression group and a low‐expression group, according to their JAB1 expression levels. The pathologists were not informed of any clinical data before the assessment.

### RNA extraction and quantitative reverse transcription‐polymerase chain reaction (qRT‐PCR)

Total RNA was extracted from cell lines and tissue samples with RNAiso (Takara Bio, Japan). mRNA expression level was determined using SYBR‐Green technology (Takara Bio, Japan) and analyzed on a Roche 480 System (Roche, Switzerland) according to the manufacturer's instructions. Relative transcript quantities were calculated using the DDCt method with β‐actin as the endogenous reference gene.

PCR primers were as follows: JAB1, 5′‐GAGATGCTCAATCAGCAGTTCCA‐3′ (forward primer) and 5′‐TCCTAAAGGCGCCAAGATTCAC‐3′ (reverse primer); β‐actin, 5′‐AGAGCCTC GCCTTTGCCGATCC‐3′ (forward primer) and 5′‐ATACACCCGCTGCTCCGGGTC‐3′ (reverse primer). This experiment was performed in triplicate.

### Western blotting (WB)

Total proteins were extracted from tissue and cell samples using the radioimmunoprecipitation assay lysis buffer. Protein concentrations were measured using the BCA Protein Assay Kit (Thermo, USA). Proteins at equivalent concentrations (40 μg) were analyzed with sodium dodecyl sulfate‐polyacrylamide gel electrophoresis before electrotransfer to a polyvinylidene difluoride membrane according to the molecular weight specified in the instructions. Following that, nonspecific antibodies were blocked with a blocking buffer containing 5% skim milk, and the membrane was placed in the primary antibody to incubate at 4°C for 10 hours. After incubation, the membrane was washed and conjugated with the HRP‐labeled secondary antibody. After that, the enhanced chemiluminescence (ECL) solution was added to the strip and an ECL detection system (Amersham Imager 680; General Electric, USA) was used for development.

### Cell counting and colony formation assays

The cell counting kit‐8 (CCK‐8; Dojindo, Rockville, MD, USA) was employed in the analysis of cell proliferation at different levels of JAB1 expression. First, approximately 5 × 10^3^ ESCC cells were counted and inoculated into a 96‐well culture plate containing 100 μL complete medium. After 0, 24, and 48 hours culture, CCK‐8 solution (10 μL) was added to each well of the plate, followed by inoculation for 1 hour. The absorbance at 450 nm was then measured with a spectrophotometer. In the plate colony formation assay, the cells were inoculated into a six‐well culture plate at the density of approximately 500 cells per well. The cells were cultured in a complete medium containing 10% FBS for two weeks and then pipetted from the medium and fixed with neutral formaldehyde. Cell colonies were counted after staining with crystal violet. Each experiment was repeated at least three times.

### Cell invasion and migration assays

In the cell migration and invasion assays, the cells were placed under serum starvation for 24 hours using a serum‐free medium before the conventional transwell migration assay. In the invasion assay, the surfaces of the transwells were coated with a thin layer (40 μL) of Matrigel (1:4; BD Biosciences San Jose, CA, USA). After that, approximately 2 × 10^5^ cells were inoculated into the upper chamber containing 200 μL serum‐free medium and placed in a 24‐well culture plate with 500 μL of 10% FBS. The migration and invasion assays lasted 24 and 48 hours, respectively. The cells were then removed from the chamber, and the upper cells wiped away while the lower ones were fixed with neutral formaldehyde, stained with crystal violet, and counted in at least three different fields of vision.

### Nude mouse xenograft assay

Six‐week‐old male BALB/c nude mice (with each group having three nude mice) were kept in a specific‐pathogen‐free (SPF) area, and each was injected subcutaneously in the right flank with 1 × 10^6^ tumor cells suspended in 100 μL PBS. Tumor growth was followed by measuring the length (i) and width (ii) of each tumor xenograft with a vernier caliper every four days. The mice were killed by CO_2_ inhalation followed by cervical dislocation 28 days later; tumors were harvested immediately for tumor weight and volume measurement. All data were retained for statistical analysis.

### Statistical analysis

The statistical analysis software SPSS 22.0 was used for building a database with the patients' clinical information, pathological features, and the experimental results. The chi‐square test was performed to define the correlations between JAB1 and relevant clinicopathological parameters. The Kaplan‐Meier estimator was used to examine the differences in five‐year and overall survival (OS) caused by JAB1 expression and to plot the survival curves. Prognostic factors were assessed through univariate and multivariate analyses. A two‐tailed *P* < 0.05 was considered statistically significant.

## Results

### JAB1 expression in ESCC

**Figure 1:**
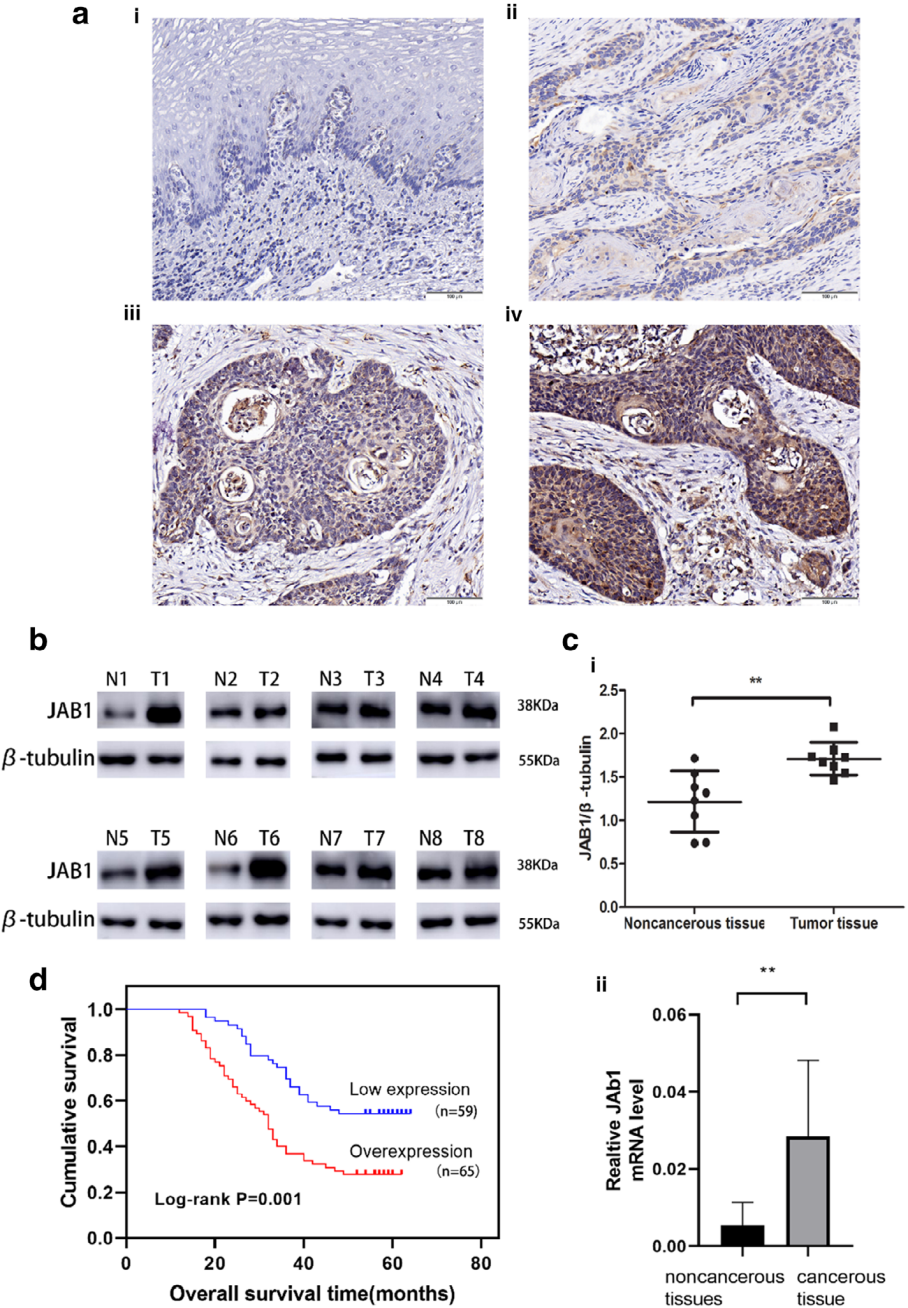
Expression of JAB1 in ESCC and its relationship with overall survival time of patients. (**a**) Immunohistochemical staining of JAB1 (magnification × 200). Scale bars, 100 μm. (**i**) Immunohistochemical staining of noncancerous tissues. (**ii**) Weak staining in cancerous tissue; (**iii**) Modern staining in cancerous tissue. (**iv**) Strong immunostaining cancerous tissue; (**b**) The bands of JAB1 and β‐tubulin in eight representative tissue sample pairs. (**c**) Quantitative analysis of JAB1 protein and mRNA in eight pairs of tissue specimens normalized to β‐tubulin (**i**) or β‐actin (**ii**). (**d**) Kaplan‐Meier analysis and log‐rank test of JAB1 for the overall survival of patients in ESCC. The results are expressed as mean ± SD. *P < 0.05; **P < 0.01; ***P < 0.001.

The IHC staining results showed that the cytoplasm of the ESCC tissue was distinctly stained positive, whereas the cytoplasm of the NAT was weakly stained (Fig 1). According to the immunohistochemical scores of 124 ESCC patients who received the Ivor‐Lewis esophagectomy, those patients were divided into a high‐expression (65, 52.4%) and a low‐expression group (59, 47.5%) (Table 1). From the RT‐PCR and WB analysis of tissues, JAB1 had a significantly higher expression level in ESCC tissue than in NAT (*P* < 0.01, Fig 1c). Eight representative pairs of protein bands on the western blot film are shown in Fig 1b.

**Table 1:** Correlations of JAB1 expression with the clinicopathological characteristics in 124 FFPe tissues of ESCC patients

		JAB1		
Parameters	Cases	Overexpression *(n* = 65)	Low expression (*n* = 59)	*P*‐value
Age (years)				0.491
≥50	102	55	47	
<50	22	10	12	
Gender				0.523
Female	28	13	15	
Male	96	52	44	
Tumor size (cm)				0.150
≥3	64	38	26	
<3	60	27	33	
T status				0.047
T1–2	59	25	34	
T3–4	65	40	25	
N status				0.050
N0	64	28	36	
N1‐2	60	37	23	
Differentiation degree				0.587
Poor	52	23	29	
Moderate‐well	72	36	36	

ESCC, esophageal squamous cell carcinoma; FFPe, formalin‐fixed paraffin‐embedded.

### High JAB1 expression may lead to early lymph node metastasis and poor prognosis in ESCC patients

At the end of the follow‐up program, 50 out of the 124 patients were still alive, resulting in an overall five‐year survival of 40.3%. The median survival was 41 months (range: 12–64). The five‐year survival rate was 27.7% in the high‐expression group and 54.2% in the low‐expression group. Kaplan‐Meier analysis was used to calculate the effects of the patients' clinicopathological characteristics on the OS rates. The log‐rank test indicated that ESCC patients with JAB1 overexpression had a markedly lower five‐year survival rate compared to the low‐expression group (27.7% vs. 54.2%, *P* = 0.001, Fig 1d, Table 2). Additionally, the multivariate analysis revealed that JAB1 overexpression was an independent prognostic risk factor for OS (*P* = 0.001, Table 2). The size or direct extent of the primary tumor (T) and degree of spread to regional lymph nodes (N) were also important prognostic factors, with high values of the T and N parameters indicating a later tumor stage and poor prognosis in ESCC patients (*P* = 0.001, Table 2). These data suggested that JAB1 overexpression was an essential factor associated with the poor prognosis of ESCC.

**Table 2:** Univariate and multivariate analyses of prognostic factors in ESCC patients

Parameters	Overall survival		
	Univariate analysis	Multivariate analysis	
	*P*‐value	HR (95% CI)	*P*‐value
Age (≥50 vs. < 50)	0.072	1.246 (0.674–2.303)	0.484
Gender (male vs. female)	0.639	1.174 (0.661–2.083)	0.584
T status (T1–2 vs. T3–4)	0.001	2.749 (1.590–4.753)	0.001
N status (N0 vs. N1–2)	0.001	2.830 (1.664–4.811)	0.001
Tumor size (≥3 vs. < 3 cm)	0.236	0.747 (0.456–1.226)	0.249
Differentiation degree (poor vs. moderate‐well)	0.407	0.746 (0.444–1.252)	0.267
JAB1 (overexpression vs. low expression)	0.001	1.677 (1.002–2.805)	0.049

ESCC, esophageal squamous cell carcinoma.

### Cell transfection

**Figure 2:**
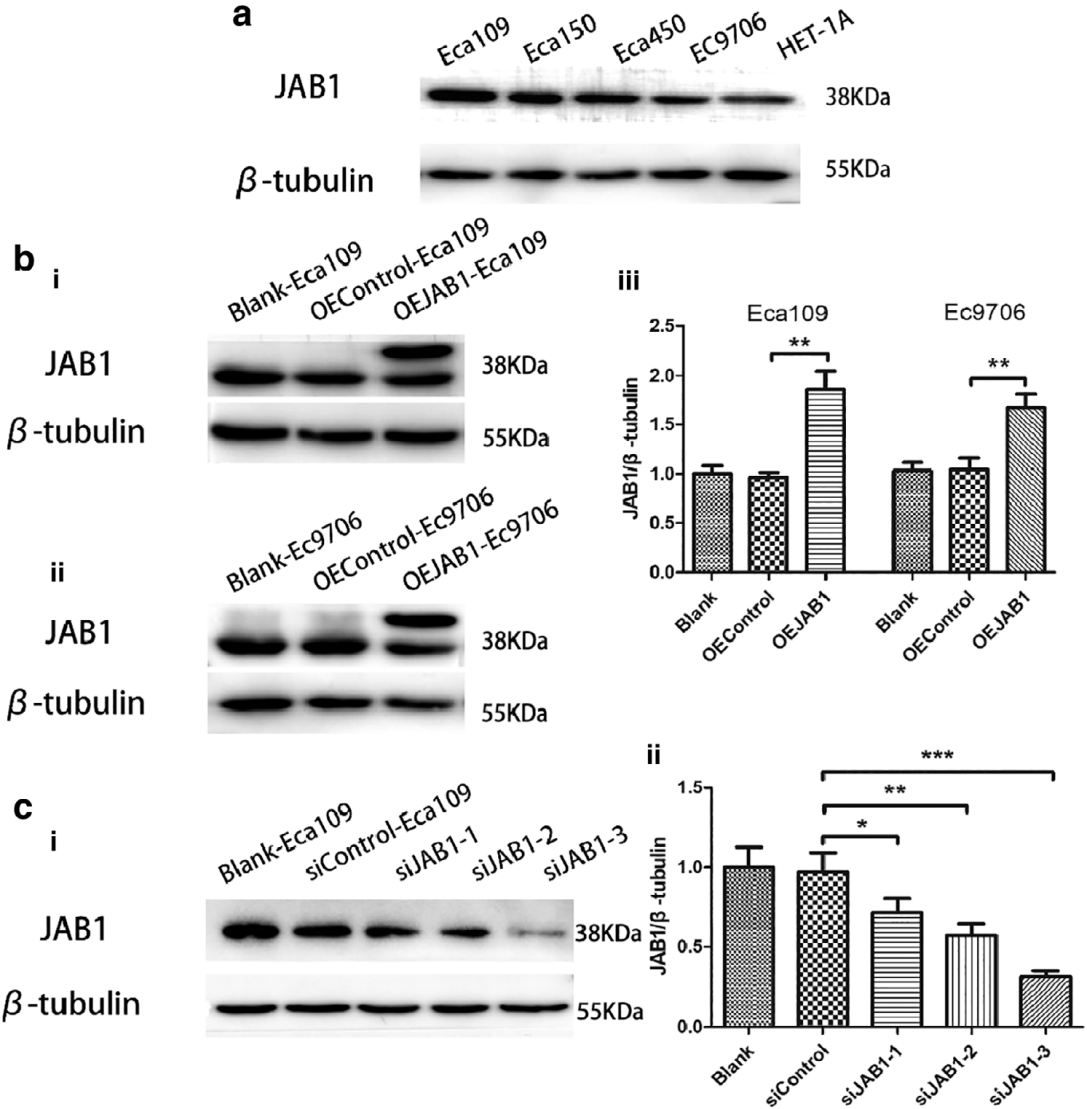
Cell selection and infection in ESCC cells. (**a**) The expression of JAB1 protein in cell lines. (**b**) The expression of JAB1 protein was overexpressed via western blot analysis. (**i** and **ii**) The bands of JAB1 and β‐tubulin in JAB1‐overexpressed Eca109 and EC9706 cells. (**iii**) Quantitative analysis of JAB1 and β‐tubulin in JAB1 determined Eca109 and EC9706 cells. (**c**) This shows (**i**) the bands and quantitative analysis of JAB1 and β‐tubulin in (**ii**) siJAB1‐1, siJAB1‐2, and siJAB1‐3. The results are expressed as mean ± SD. *P < 0.05; **P < 0.01; ***P < 0.001.

Western blot was used to detect and analyze JAB1 expression in the four ESCC and the HET‐1A cell lines. The results revealed that JAB1 overexpression was extensive in the ESCC cell lines compared to the HET‐1A cell line (Fig 2a). JAB1 expression in Eca109 and EC9706 was upregulated and downregulated using lentivirus and siRNA, respectively. The knockdown group was further divided into a blank control, and three siControl subgroups that were transfected with SiJAB1‐1, SiJAB1‐2, and SiJAB1‐3, respectively. Through western blotting, it was found that JAB1 expression was sharply downregulated in the siJAB1‐3 group (Fig 2c). Therefore, the corresponding sequence was used in subsequent experiments. Likewise, compared to the blank control, JAB1 overexpression was significantly higher in the lentivirus‐transfected ESCC cell lines (Fig 2b).

### JAB1 promoted ESCC cell growth

**Figure 3:**
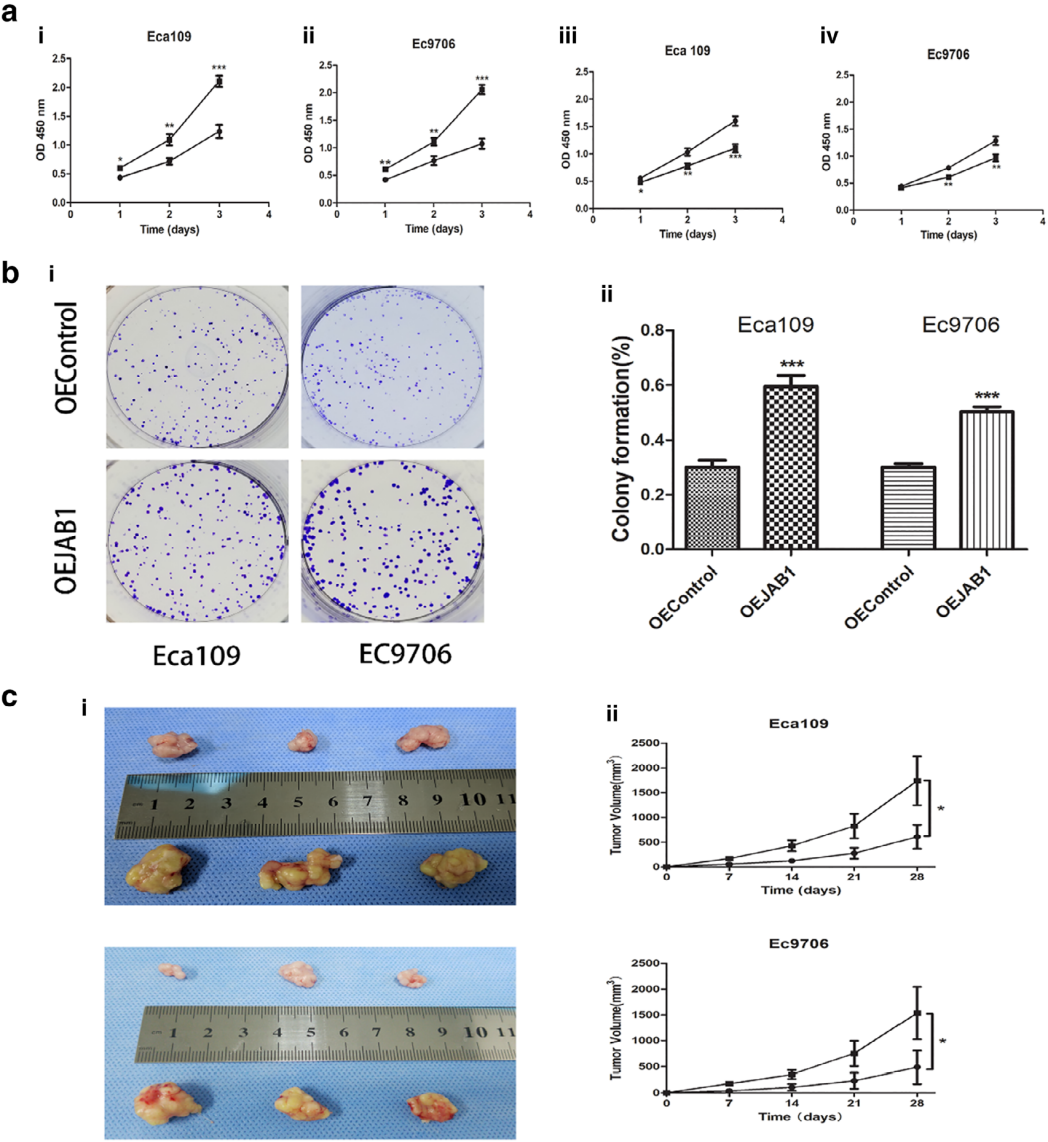
JAB1 promotes the proliferation of ESCC cells. (**a**) The viability of JAB1‐overexpressed (**i** and **ii**) or JAB1‐knockdown (**iii**) cells was measured with the CCK‐8 method. (**i**, **ii**) (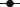) OEControl and (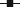) OEJAB1. (**iii**, **iv**) (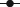) siControl and (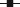) siJAB1‐3. (**b**) Clonogenic assays were applied to evaluate the effect of JAB1 on cell growth. The images of colony formation are shown (**i**). (**c**) The tumorigenesis ability of JAB1 in vivo was measured with xenografted tumor models (**i**). The tumor volumes were markedly changed with JAB1 overexpression (**ii**). The results are expressed as mean ± SD. *P < 0.05; **P < 0.01; ***P < 0.001. Eca109 (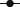) OEControl and (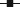) OEJAB1. Ec9706 (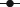) OEControl and (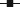) OEJAB1.

The CCK‐8 assay revealed that the Eca109 and EC9706 cells transfected with the JAB1 overexpression lentivirus showed significantly increased absorbance compared to normal cells, (Fig 3a). This indicated that high JAB1 expression in ESCC cells promoted cell growth. Meanwhile, cell growth became slower in the ESCC cell lines with downregulated JAB1 expression after transfection with the siRNA. Clonogenic assays showed that JAB1 upregulation enhanced the colony‐formation ability of Eca109 and EC9706 cells (*P* < 0.001, Fig 3b).

### Effect of JAB1 on ESCC cell migration and invasion via the EMT

**Figure 4:**
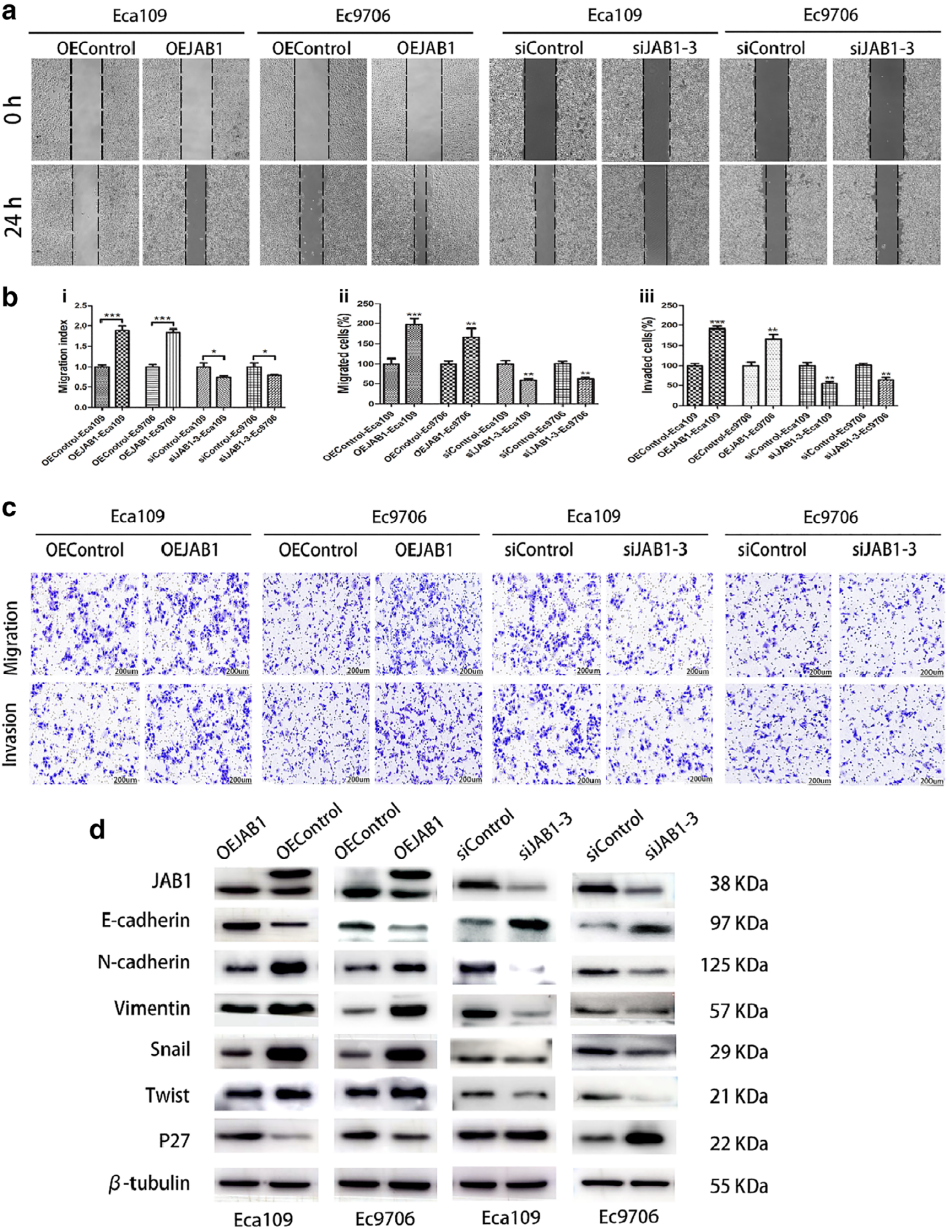
JAB1 is essential for the proliferation, migration and invasion of ESCC cells and molecular markers. (**a**) Representative images and quantification analysis (**b**) of a wound‐healing assay of the indicated ESCC cells. (**i**) The migration index represents migration speed in relation to the control group. (**b** and **c**) Representative images and quantification analysis of (**ii**) migratory or (**iii**) invasive behavior of the indicated ESCC cells. Scale bars, 200 μm. (**d**) Western blotting demonstrated that the expression of EMT‐related markers (E‐cadherin, N‐cadherin, Vimentin, Snail and Twist) and P27 was altered with the dysregulated JAB1. The results are expressed as mean ± SD. *P < 0.05; **P < 0.01; ***P < 0.001.

The transwell assay revealed that the Eca109 and EC9706 cells with upregulated JAB1 expression displayed enhanced cell migration and invasion, whereas the downregulation group showed the opposite results (Fig 4b). The difference in cell migration was statistically significant. In the scratch wound healing assay, the JAB1 upregulation group showed a faster speed of cell migration (Fig 4a). These data indicated that JAB1 expression had an impact on ESCC cell migration and invasion.

Additionally, it was noted that the relevant EMT indicators (E‐cadherin, N‐cadherin, twist, Vimentin, and Snail) were affected by JAB1 expression in the ESCC cell lines. When JAB1 expression in the Eca109 and EC9706 cell line was downregulated, the expression level of E‐cadherin, an epithelial cell marker, was remarkably elevated, whereas mesenchymal marker expression (e.g., N‐cadherin, Vimentin, Snail, and twist) was reduced (Fig 4d). In contrast, the upregulation of JAB1 expression in the Eca109 and EC9706 cell lines promoted the expression of N‐cadherin, Vimentin, Snail, and twist and inhibited the expression of E‐cadherin (Fig 4d).

### Faster tumor growth in ESCC xenograft models with upregulated JAB1 expression

Considering the complexity of metastatic tumor growth in ESCC patients, this study used BALB/c nude mice to construct ESCC xenograft models and simulate the process of metastatic tumor growth. The nude mice were fed for 28 days, during which the metastatic tumors in the JAB1 upregulation group obviously outgrew those in the control group (*P* < 0.05, Fig 3c).

## Discussion

Esophageal squamous cell carcinoma is a common, highly aggressive tumor with frequent invasion and inferior prognosis. Surgical resection is considered to be the best treatment for early and localized ESCC.^5^ In the past, the primary predictor of surgical outcome was the TNM stage of the primary tumor. Nevertheless, it has been found that many patients who had earlier TNM stages would undergo disease recurrence, metastasis, and eventually die within five years following surgery. In contrast, other patients in the advanced stage achieved long‐term survival after treatment, suggesting that the TNM staging was inadequate in predicting the prognosis of patients. Thus, there is a need to seek sensitive, efficient, and convenient molecular biological indicators to supplement TNM staging. Many indicators, such as EGFR/HER‐2/VEGF, have been extensively studied in ESCC.^19^, ^20^ However, so far, none of those indicators have been recognized as a predictor of the prognosis of ESCC patients. At present, the proposed problem remains a hot research topic worldwide.

It has been shown in many studies that high JAB1 expression exists in a variety of malignant tumors, such as head and neck squamous cell carcinoma, cholangiocarcinoma, and colon carcinoma.^21^, ^22^, ^23^, ^24^ Also, JAB1 expression in tumor tissue is closely associated with the degree of malignancy, which makes JAB1 a promising prognosis prediction tool.^22^, ^25^ However, there is currently inadequate research on JAB1 in ESCC. Previous studies have reported that JAB1 function and its possible mechanism may be related to the prognosis of cancer patients;^18^, ^21^, ^22^ however, it has not been thoroughly explored in ESCC. The present study focused on the expression, function, and possible mechanism of JAB1 in ESCC, associated with the establishment of the corresponding virus‐transfected cell line model and animal models of metastasis. The advantages of the present study lie in its extensive scope and comprehensive research.

In this study, ESCC specimens collected from 124 patients were analyzed. It was found that JAB1 was highly expressed in cancer tissues of ESCC patients, and the expression level was significantly correlated with the prognosis of ESCC patients. Previous research by Wang *et al*. has shown that JAB1 promotes the progression of malignancies.^26^ This study revealed JAB1 overexpression in different ESCC cell lines and the role of JAB1 overexpression in ESCC progression as a promoter of cell growth.^22^, ^25^ In animal experiments, we found that JAB1 overexpression made tumors of ESCC xenograft models grow faster. In the EMT process, cancer cells lose their epithelial features and acquire characteristic mesenchymal features, which is considered a critical process in the metastasis of tumor cells, as well as in the spread of malignant tumors. Many previous studies have shown that the EMT indicators are an essential factor affecting tumor metastasis.^12^, ^13^, ^14^, ^15^ We found that changes in JAB1 expression had a significant impact on the expression of various EMT indicators, which further affects ESCC metastasis via the EMT process.

This study also noted that JAB1 affected other oncogenes in ESCC and induced changes that might collectively influence the prognosis in ESCC patients. p27 is a regulator of the cell cycle and a cyclin‐dependent kinase inhibitor.^23^, ^27^, ^28^, ^29^ Reduced expression of the p27 protein is an independent prognostic marker in a large variety of cancers and is associated with an unfavorable prognosis. Changes in JAB1 expression affected the expression of a variety of antioncogenes in ESCC (Fig 4), which provided evidence for the impact of JAB1 on the degree of malignancy of ESCC in multiple directions and through a range of pathways.

Currently, the treatment of ESCC is a challenge in the field of medicine, and multiple factors affect the prognosis of ESCC patients. Our research was unable to comprehensively reveal the entire mechanism affecting the prognosis of ESCC patients. This study was conducted using tissue, cell, and clinical information, and also measured gene upregulation and downregulation, cell function and animal experiments, reflecting the width and depth of our study. While this research systematically studied the properties, functions, and roles of JAB1 in ESCC, some of its limitations included the retrospective design and inevitable bias in the selection of patients. Furthermore, a number of factors have a tremendous influence on the prognosis of patients, including whether patients were given postoperative adjuvant therapy, such as radiotherapy and chemotherapy, as well as how adjuvant therapy was administered. The effect of adjuvant therapy on the prognosis of patients deserves further discussion and study.

In conclusion, overexpression of JAB1 promoted the proliferation, migration, and invasion of ESCC cells, and was significantly associated with poor prognosis of ESCC patients. We identified JAB1 as a potential biomarker to predict the prognosis of ESCC patients and a direction for promising targeted therapy. Many patients with high JAB1 expression are expected to benefit from postoperative adjuvant therapy.

## Disclosure

No authors report any conflict of interest.
